# PSI-Guided Mandible-First Orthognathic Surgery: Maxillo-Mandibular Position Accuracy and Vertical Dimension Adjustability

**DOI:** 10.3390/jpm11111237

**Published:** 2021-11-21

**Authors:** Giovanni Badiali, Mirko Bevini, Ottavia Lunari, Elisa Lovero, Federica Ruggiero, Federico Bolognesi, Liliana Feraboli, Alberto Bianchi, Claudio Marchetti

**Affiliations:** 1 Department of Maxillo-Facial Surgery, IRCCS Azienda Ospedaliero-Universitaria di Bologna, 40138 Bologna, Italy; giovanni.badiali@unibo.it (G.B.); federico.bolognesi5@unibo.it (F.B.); ferabolililiana@gmail.com (L.F.); claudio.marchetti@unibo.it (C.M.); 2 Department of Biomedical and Neuromotor Sciences, University of Bologna, 40126 Bologna, Italy; ottavia.lunari@studio.unibo.it (O.L.); elisa.lovero@unibo.it (E.L.); ruggierof.md@gmail.com (F.R.); 3 Department of Surgery and Surgical Specialties, Azienda Ospedaliero-Universitaria Rodolico-S. Marco, University of Catania, 95124 Catania, Italy; alberto.bianchi@unict.it

**Keywords:** orthognathic surgery, patient-specific implants, mandible-first, CAD/CAM, virtual surgical plan

## Abstract

In orthognathic surgery, patient-specific osteosynthesis implants (PSIs) represent a novel approach for the reproduction of the virtual surgical planning on the patient. The aim of this study is to analyse the quality of maxillo-mandibular positioning using a hybrid mandible-first mandibular-PSI-guided procedure on twenty-two patients while the upper maxilla was fixed using manually bent stock titanium miniplates. The virtual surgical plan was used to design PSIs and positioning guides, which were then 3D printed using biocompatible materials. A Cone Beam Computed Tomography (CBCT) scan was performed one month after surgery and postoperative facial skeletal models were segmented for comparison against the surgical plan. A three-dimensional cephalometric analysis was carried out on both planned and obtained anatomies. A Spearman correlation matrix was computed on the calculated discrepancies in order to achieve a more comprehensive description of maxillo-mandibular displacement. Intraoperatively, all PSIs were successfully applied. The procedure was found to be accurate in planned maxillo-mandibular positioning reproduction, while maintaining a degree of flexibility to allow for aesthetics-based verticality correction in a pitch range between −5.31 and +1.79 mm. Such a correction did not significantly affect the achievement of planned frontal symmetry.

## 1. Introduction

The mandible-first approach for orthognathic surgery is an alternative procedure to the more widespread maxilla-first approach, which carries an array of theoretical advantages, such as reducing the mandibular condylar sag and reducing the strain on the upper maxillary miniplates while carrying out the mandibular Bilateral Sagittal Split Osteotomy (BSSO), subsequently improving the quality of planning reproduction [1].

While part of the theoretical advantages has not been demonstrated yet, the increased intraoperative flexibility offered by this approach in terms of vertical correction is counterbalanced by its reliance on the exactness of the post-osteosynthesis spatial relationship between proximal and distal mandibular segments.

In the pursuit of a technique which could reliably reproduce the digitally planned spatial relationship between the mandibular condyle-bearing segments and teeth-bearing segment, we combined the mandible-first approach with a PSI-guided mandibular procedure and demonstrated that it leads to a satisfactory reproduction of the planned mandibular anatomy [2].

As current literature on mandibular and bimaxillary PSI-guided orthognathic surgery is still scarce, the potential failure of the procedure due to dental interferences caused by combined maxillary and mandibular inaccuracies—beyond the surgeon’s level of control—has been reported but has not been extensively investigated [3,4]. The approach we propose should also theoretically limit such combined inaccuracies, transferring them from the occlusal surface, where they are most impactful, to the maxillary osteosynthesis surface, which can accept and dampen such minimal inaccuracies, rendering them mostly negligible.

To further analyse the proposed technique, we determined the surgically achieved positional accuracy of both the upper maxilla and mandibular teeth-bearing fragment, in relation to the cranial base, on a cohort of patients treated with this approach.

In this paper, we present our evaluation of the approach itself in terms of precision and flexibility allowing for aesthetics-based intraoperative verticality correction, also assessing whether such correction could lead to other unwanted displacements of the skeletal segments analysed.

We also focused on the achieved frontal symmetry and the correction of the yaw component of asymmetries, which is the most difficult to control in traditional splint-guided surgery [5].

## 2. Materials and Methods

Twenty-two patients undergoing orthognathic bimaxillary surgery at the Oral and Maxillofacial Surgery Unit of the Sant’Orsola-Malpighi University Hospital (Bologna, Italy) between July 2017 and June 2019—an equal number of males and fifteen females, mean age 26 years (range 18–43 years)—were included in the trial. Eight patients were diagnosed with skeletal class II deformity (one with combined facial asymmetry), ten were diagnosed with skeletal class III (six with combined asymmetry), three patients were diagnosed with class I facial asymmetry and one with anterior open bite. The present protocol was approved by the Sant’Orsola-Malpighi University Hospital ethics committee (approval number 238/2012/0/Disp PL02, amended 18 October 2016); the study conformed to the principles of the Declaration of Helsinki. Written informed consent was obtained from all the patients upon enrolment to the trial [2].

### 2.1. Case Planning and Surgery

One month prior to surgery all patients underwent a pre-operative CBCT-scan (NewTom VGI Evo, Cefla Group, Imola, Italy), (24 × 19 cm FOV, 0.3 mm voxel) in a clinically determined natural head position and using a wax bite obtained in clinically set condylar centric relation.

Contextually, dental digital models were acquired using the CS 3600 intraoral scanner (Carestream Health Inc., Rochester, NY, USA), and 3D printed using a stereolithographic printer (Form 2, Formlabs Inc., Somerville, MA, USA). The final occlusion was determined on the stereolithographic models and transferred to the digital models via re-scan [2].

**Figure 1:**
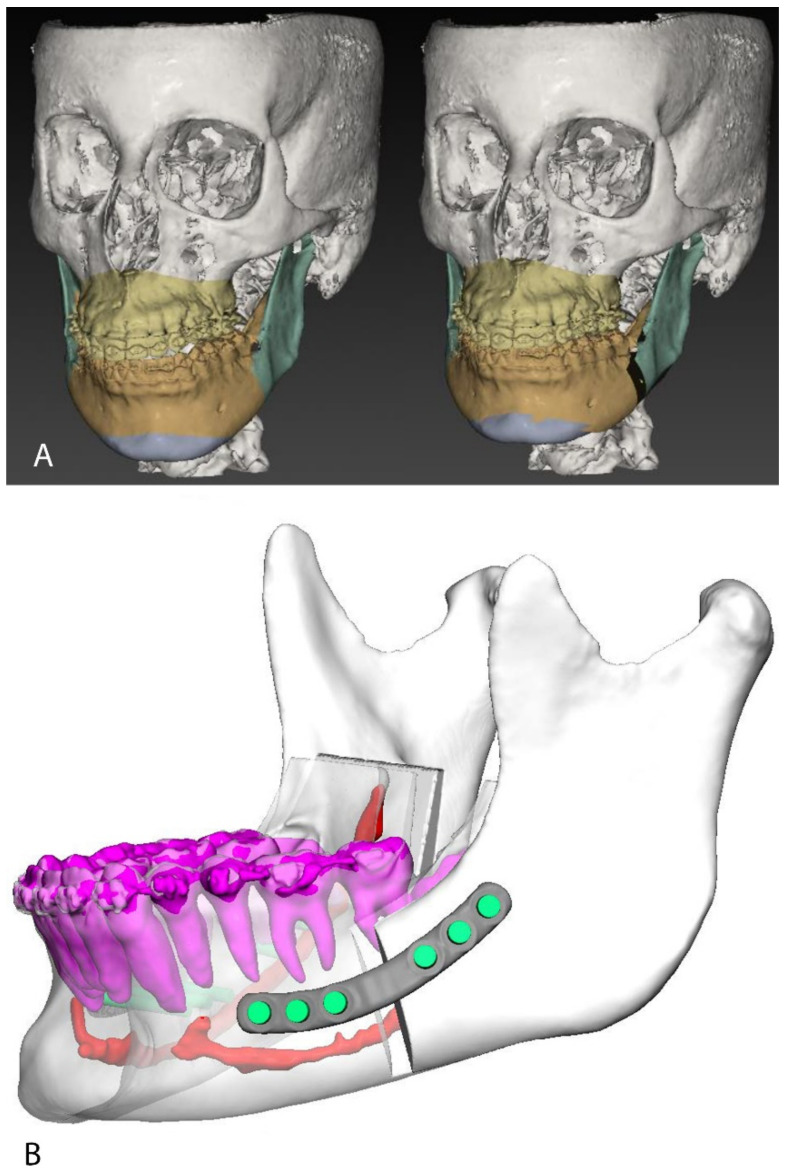
(**A**): Virtual surgery planning in IPS CaseDesigner (v1.4.4.1, KLS Martin, Tuttlingen, Germany, www.klsmartin.com/en/products/individual-patient-solutions-cmf/ips-casedesigner/ accessed on the 1 October 2021). (**B**): Patient-specific plates CAD design. Teeth roots and inferior alveolar nerves were segmented and carefully avoided while planning the plates’ position and screw trajectories.

IPS Case Designer software (KLS Martin, Tuttlingen, Germany) was used to perform a three-dimensional cephalometry according to Swennen et al. [6] and plan the surgical skeletal movements (Figure 1A). Proximal and distal mandibular segments were positioned in order to reduce interference along the osteosynthesis surface and minimize discontinuity of the inferior border.

Intermediate and final surgical splints were designed according to the virtual surgical plan. Mandibular autorotation to avoid interferences in the construction of the intermediate surgical splint was set using the dedicated function of IPS CaseDesigner.

Although the mandibular procedure was designed as a potentially splint-less surgery, intermediate surgical splints were manufactured as a back-up solution in case of intraoperative failure of the system. Eventually, the intermediate splint was systematically used to stabilize the teeth-bearing fragment during fixation.

On the basis of the VSP, KLS Martin biomedical engineers designed the individualized mandibular PSI-positioning guides, patient-specific plates, and splints under the surgeon’s suggestions (Figure 1B). The positioning guides were designed to guide the buccal and sagittal osteotomies, as well as the cranio-caudal level of the lingual osteotomy to reproduce the digitally designed Bilateral Sagittal Split Osteotomy (BSSO). Screw trajectories were planned in order to avoid dental roots and the path of the inferior alveolar nerve (Figure 1B). Three guide types were used throughout the trial and were compared in our previous work on this approach [2].

The custom 3D printed titanium alloy (Ti6Al4V) plates were designed to fixate the proximal and distal mandibular fragments in their planned positions using the screw fixation holes, drilled according to the positioning guides, as reference. Splints were manufactured using 3D printed dental resin. Digital three-dimensional models of bony fragments and plates were also provided by KLS Martin (in .STL format) for outcome evaluation.

**Figure 2:**
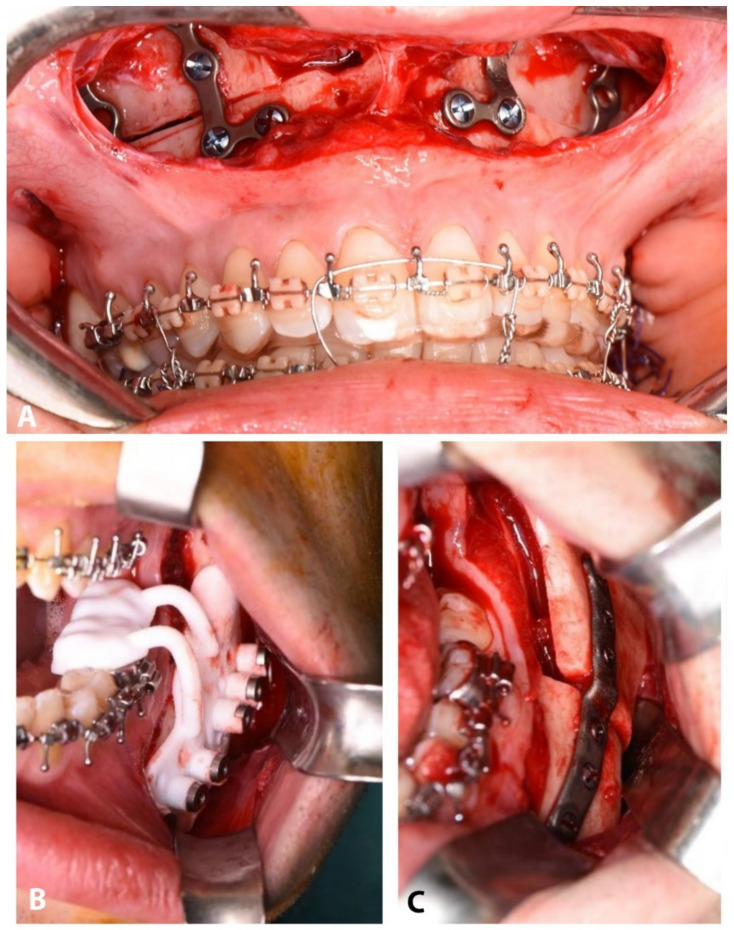
Intraoperative view of maxillary manually bent plates (**A**), mandibular positioning guide (**B**), and mandibular CAD/CAM plate (**C**).

A single surgeon (G.B.) operated on all patients by means of mandible-first approach, using the PSI system (Figure 2B,C). The mandibular bony surface was exposed through the conventional vestibular incision to perform BSSO. Two titanium screws were used to secure the guide to the mandible using the designed fixation holes (1.5 mm) to avoid mobilization while performing the osteotomy. According to the guide, the surgeon marked the osteotomy lines using ultrasonic bone-cutting tools (Piezo-Surgery, Mectron, Cerasco, Italy) and drilled the transfer holes for the plate using an Angulus2 angulated drill (KLS Martin, Tuttlingen, Germany). The guide was then removed to complete the osteotomy. The condyle-bearing and the teeth-bearing fragments were fixed in the planned position using the pre-drilled transfer holes (2.0 mm) to position the PSIs. The intermediate splint was positioned to secure the mandibular teeth-bearing fragment in position while performing the osteosynthesis to facilitate the procedure. The upper maxilla was then fixed in the best aesthetics-based vertical position using standard manually bent titanium miniplates and 2.0 mm screws, under the guide of the final splint (Figure 2A).

### 2.2. Outcome Analysis

A CBCT scan was performed on all patients one month after surgery before any tooth movements occurred, thanks to rigid orthodontics and daily use of the final splint. The same machine and parameters of the pre-operative scan were used; the occlusion was kept at maximum intercuspation. The post-operative CBCT scan was segmented to obtain a 3D model of the post-operative skull, mandible, and mandibular plates using DICOM to Print software (3D Systems, Rock Hill, SC, USA) and exported in STL format [2].

Two analyses were carried out: rigid body transformation and 3D cephalometry.

**Figure 3:**
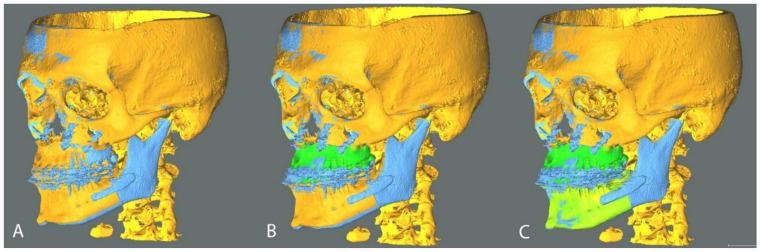
Analysis method in CloudCompare software (v2.9.1, www.cloudcompare.org, accessed on the 1 October 2021). Planned models (orange) were aligned to postoperative models (blue) on the basis of the cranial base (**A**). Planned upper maxilla was aligned to postoperative maxillary position and the transformation was recorded; the alignment was visually checked via generation of colorimetric surface maps (**B**); An analogous protocol was applied to the mandibular teeth-bearing fragment (**C**).

The positions of planned and post-operative STL objects were compared using the open-source software CloudCompare (CloudCompare Project, www.cloudcompare.org, accessed on the 1 October 2021). The method is based on the OrthoGnathicAnalyzer [7], to which we applied Iterative Closest Point (ICP) alignments to reduce operator-dependence. In order to fix a frame of reference, the planned and obtained cranial base models were superimposed via an ICP alignment (Figure 3A). The alignment was then visually checked by means of colorimetric surface maps inspection. The discrepancy between planned and obtained post-operative position of the mandibular teeth bearing fragment and upper maxilla were evaluated by analyzing the displacement in terms of rotation (roll, pitch and yaw angles) and translation (antero-posterior, lateral and vertical). These movements were determined aligning the planned model to the post-operative result via ICP alignment and checking the obtained alignment via colorimetric map inspection (Figure 3B,C).

The rigid body transformation coordinates system was set to originate at the centre of the bounding box of each considered model. The roto-translational transformation the planned model had to undergo to be aligned to the post-operative model is representative of the intraoperative error. The rigid body transformation matrix representing this shift and rotation was then evaluated, displaying the transformation according to the more intuitively comprehensible Euler angles convention [2].

The signed discrepancies were tabulated applying conventional signs. Positive signed values identify forward, upward and left lateral translations; a positive pitch angle identifies a clockwise rotation as seen from the patient’s right lateral aspect; a positive roll angle identifies a clockwise rotation as seen from in front of the patient; a positive yaw angle indicates a clockwise rotation as seen caudally to the patient.

Two further parameters were defined to comprehensively describe the angular and translational displacement of each 3D model considered: total angular error and total translational error. The first is the angle in the axis-angle representation of a rigid body transformation, while the second is the module of the translation vector. Both measures are always positive by definition and were used to avoid positive and negative displacements canceling each other on average in the description of displacement [2].

A three-dimensional cephalometric analysis was performed on planned and postoperative 3D models. The considered cephalometric data is shown in Table 1.

### 2.3. Statistical Analyses

All measures were summarized using median and interquartile range (IQR), due to the limited sample size (*n* = 22). However, to allow for comparison with the existing literature, mean and standard deviation were also provided for each measure.

In order to assess how the intraoperative surgical plan correction impacted on the symmetry if the result and overall precision, a Spearman correlation matrix (*n* = 22) was calculated for each considered rotation, translation, and cephalometric plan to post-op difference. Rotational and translational discrepancies between VSP and postoperative results were considered both as signed and absolute values.

Non-parametric two-tailed tests were used in all cases. The significance level was set to α = 0.05 for all tests. IBM SPSS Statistics 25 (IBM, Armonk, NY, USA) was used to perform the analyses.

## 3. Results

The average follow-up is 22 months (range 13–37). Descriptive statistics are reported in Table 2 and Table 3.

### 3.1. Rigid Body Transformation Outcomes

In the maxillary segment positioning, we obtained a median total angular error of 2.31° (IQR 1.41°) and a median total translational error of 2.03 mm (IQR 1.83 mm). With the exception of the pitch angle (median absolute value 1.51°), the alteration of which is compatible with the intraoperative verticality correction based on the autorotation of the mandible, median absolute value rotational shifts were around 0.75° from planned (roll 0.78°, yaw 0.72°).

Translational median discrepancies were all within 1.5 mm in absolute value from planned. The greatest discrepancy can be found in anteroposterior translation, with a median of 1.39 mm in absolute value, and a tendency to retrusion, with a signed median value of −1.27 mm.

In the mandibular teeth bearing fragment positioning, the median total angular error was 2.34° (IQR 1.30°) and the median total translational error was 2.01 mm (IQR 1.34 mm). Similarly to the upper maxilla, the median pitch angle was 1.2° in absolute value, with yaw and roll both 0.75° in absolute value. Median absolute value translations were also around 1 mm (lateral = 0.93 mm, antero-posterior = 1 mm, vertical = 0.70 mm)

Results obtained from class II and class III patients were tabulated according to the skeletal class, and an inter-class comparison via Mann–Whitney test was carried out. Comparing the maxillary and mandibular antero-posterior displacement were the most divergent value (class III: avg. −0.75 mm, st.dev 1.42 mm; class II: avg. −2.31 mm, st.dev 2.39 mm) however this difference did not reach statistical significance, at *p* = 0.109, as all of the other compared values.

**Figure 4:**
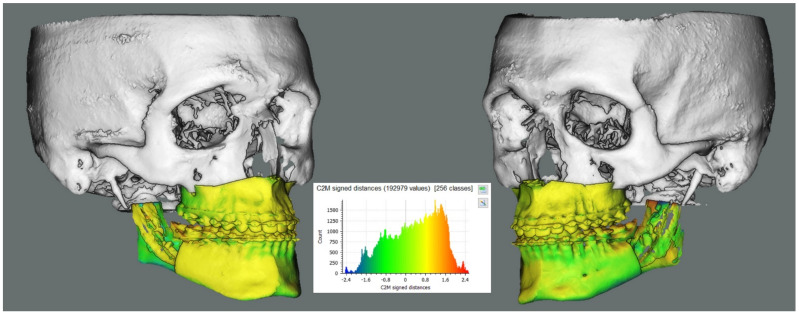
Simulation of the obtained median maxillary and mandibular displacements on an example surgical plan in CloudCompare software (v2.9.1, www.cloudcompare.org, accessed on the 1 October 2021). A colorimetric surface map was used to show surface differences of the simulated median position from planning. Rami were not included in the simulation in order to increase the visibility of the teeth-bearing fragment. An analogous simulation for the rami position relative to the teeth-bearing fragment is shown in our previous work on this cohort [2].

Median maxillary and mandibular roto-translations obtained are simulated on an example case and a colorimetric surface map between planned and simulated shifted position was calculated. The result is shown in Figure 4.

### 3.2. Cephalometric Outcomes

The frontal symmetry cephalometric measures considered in absolute value yielded median results below 1.5 mm on midline measures (A/Sag, B/Sag, Pog/Sag, UIs/Sag and LIs/Sag) and on the Δ Go/sag. The largest median discrepancy was obtained in the Δ U3/Sag at 2.78 mm, while the median Δ U6/Sag was 1.65 mm.

### 3.3. Correlation Outcome

Spearman’s correlation coefficients deemed clinically significant were extrapolated from the correlation matrix and reported in Table 4.

All following reference to cephalometric measurements is to be intended as difference between planned and post-op values.

Strong positive correlations were found between analogous translational and rotational values of upper maxilla and mandibular teeth-bearing fragment, ranging from 0.523 with *p* = 0.012 for pitch to 0.819 with *p* < 0.001 for anteroposterior translation.

A-McNamara and Incisal Protrusion are negatively correlated (−0.519, *p* = 0.013) as expected in a situation in which antero-posterior discrepancy is partly due to intraoperative modification of the verticality. This finding is also supported by the correlation between mandibular pitch and maxillary antero-posterior translation (−0.542, *p* = 0.009).

Notably, no significant correlations were found between symmetry cephalometric indicators (Maxillary Deviation, Mandibular Deviation, Mental Deviation, UIs/Sagittal Plane, Lis/Sagittal plane, U3/Sagittal Plane and U6/Sagittal Plane) and pitch angular displacement or vertical translation.

## 4. Discussion

The debate between maxilla-first and mandible-first approach has been ongoing for decades and, although the maxilla-first approach is more widely used, the mandible-first approach is preferable in a range of cases, for the fact that it provides a more stable frame of reference by using the upper maxilla as a guide. Additionally, executing the BSSO first avoids the stress caused on the miniplates used for maxillary fixation, and theoretically reduces the condylar displacement caused by the supine position of the patient and anesthesia. However, as Borba et al. state in their systematic review on the matter, little data is available to support the greater part of these claims [1].

Although the use of PSIs in maxillary surgery is already well documented [8,9,10,11], their use in mandibular orthognathic surgery is less frequently reported, mainly due to the difficulty of obtaining a stable reference for plate fixation on the lateral mandibular aspect [3,4,12,13].

Li et al. [3] reported satisfactory precision results obtained on a cohort of patients using bimaxillary PSIs (*n* = 10): this procedure requires minimal to no planning error with flawless subsequent execution of said plan and allows no intraoperative plan correction. A potential result of even minimal inaccuracies introduced in the above-mentioned approach are skeletal and dental interferences, possibly ending in PSI inapplicability.

Suojanen et al. [4], in a cohort study of 30 patients, reported a significant percentage of cases in which PSIs were inapplicable or needed modification. More recent studies, however, do not report this occurrence, although examining smaller cohorts of patients.

Furthermore, the rigidity and relative brittleness of sintered titanium alloy makes the modification of custom-made implants almost impossible, needing to revert to stock miniplates and/or bicortical screws in case of PSI inapplicability.

An additional factor affecting the adoption of PSI-guided approaches is cost of PSIs which, at the time of writing, amounts to three times the stock titanium miniplates. Double-jaw PSI solutions are subsequently significantly more expensive than single-jaw PSIs

Published data on PSI-guided mandibular orthognathic surgery suggests that the approach we are hereby presenting has never been proposed before by other authors. As per our previous findings on this approach [2], this procedure yielded an accurate mandibular anatomy reproduction.

A more thorough planning, when compared to empiric proximal fragment positioning, is needed to avoid interferences between the bony segments along the BSSO osteosynthesis surface; interferences in this region could in turn lead to condylar displacement in the glenoid fossa.

From a clinical point of view, the aesthetic result was positive in all patients, part of which is due to the intraoperative plan correction in which the modification of soft tissue could be accounted for in real time. Plate infections (4.5%) are on par with the rate observed in patients in which conventional titanium plates were used [14].

The overall accuracy of the procedure, also taking into account the vertical correction, is highly satisfactory. Although the median absolute value mandibular autorotation was only 1.51°, giving a median verticality correction of 0.93 mm in absolute value, no correlation between frontal symmetry shift measures and pitch or vertical translation was found in an autorotation range between −4.03° and +2.24°, allowing for a vertical plan correction between −5.31 mm and +1.79 mm.

When comparing our obtained precision results with a recent meta-analysis on the precision results of digitally planned orthognathic surgery [15], the results we obtained are, on absolute value average, slightly more precise than the ones reported in literature (pitch = 2.75°, yaw = 1.7, and roll = 1.1). Maxillo-mandibular pitch is reported as the most divergent measurement, due to intraoperative autorotation-based verticality correction. Notably, the median absolute value yaw discrepancy in our cohort is around 0.75°, while such measurement is reported by the meta-analysis as 1.7° on average, which is more than double what we obtained.

When analysing the single studies upon which such meta-analysis is based, together with other similar studies [16,17,18,19], the variety of analysis methods used is ample, and a direct comparison with our study cannot be carried out. Our group is subsequently prospecting a future study to properly investigate the actual increase in precision of PSI-based procedures, while keeping biases and methodological differences at a minimum.

Our interpretation of the obtained maxillary translational values suggests that a quota of antero-posterior shift (median −1.27 mm) could partly be a by-product of the verticality correction via autorotation (mandibular pitch and maxillary antero-posterior translation are correlated with a coefficient of −0.542 and *p* = 0.009) and partly to be attributed to a quota of condylar sag or displacement in the fossa, due to the supine position of the patient and myorelaxation. This displacement, however measurable, was not clinically significant and did not impact on the resulting occlusion. This finding was also present in other authors’ work, albeit it appears that in our cohort the extent of this retroposion is of a slightly lesser extent [16,17].

Interestingly, the antero-posterior maxillary displacement was the most different parameter when comparing class II and class III patients, with class II patients in our cohort having an increased tendency to maxillary retroposition, possibly due to an increased posteriorly directed muscular tension and a more condylar sagging-prone anatomy. This difference, however, did not result statistically significant at the inter-class comparison via Mann–Whitney test (*p* = 0.109). This may be due to the small sample size of the class-coherent subgroups, and further results may be needed to confirm this finding.

The correlation values according to which the upper maxilla and the mandibular teeth-bearing fragment moved in a coordinated fashion further the notion of accuracy of CAD-CAM splints, in this case mainly the final splint.

All in all, the authors’ opinion is that PSI-guided surgery should not be synonym of splintless surgery, Given the limited cost of 3D printed splint production, splints should be produced with the triple function of teeth-bearing segment stabilizers, backup solution and additional plan transfer-checking method also in PSI-guided cases.

## 5. Conclusions

Following our previous work on the matter, the findings above support the hypothesis that the proposed mandible-first mandibular PSI-guided procedure yields a positive outcome without sacrificing adjustability and thus reducing the risk of PSI inapplicability.

Vertical intraoperative plan correction through mandibular autorotation does not correlate with midline shift, allowing for a safe vertical correction ranging between −5.31 and +1.79 mm, although in most cases there was no need for intraoperative plan correction, and the plan was accurately transferred to the patient as previously designed. Notably, this procedure achieved an accurate correction of the yaw component of asymmetries, the least controllable in splint-guided surgery. Overall, the adoption of single-jaw PSIs could be considered a viable and more economically sustainable alterative to double-jaw PSIs, while retaining the advantage of precisely guiding the condyle-bearing segments in the planned position without sacrificing flexibility.

## Figures and Tables

**Table 1:** Considered cephalometric measurements and relative description.

Considered Cephalometric Measurements	
A/Sag	Distance of point A from the sagittal plane (maxillary deviation)
B/Sag	Distance of point B from the sagittal plane (mandibular deviation).
Pog/Sag	Distance of pogonion from the sagittal plane (mental deviation).
UIs/Sag	Distance from upper incisors midpoint to sagittal plane.
LIs/Sag	Distance in millimetres from Lower Incisors midpoint to Sagittal plane.
△Go/Sag	Difference between left and right gonion to sagittal plane distances in millimetres.
△U3/Sag	Difference between left and right upper canine to sagittal plane distances.
△U6/Sag	Difference between left and right first upper molars to sagittal plane distances.
A/McNamara	Distance of point A from McNamara plane.
Incisal Protrusion	Distance in millimetres of the upper incisors’ midpoint to plane A (plane parallel to McNamara plane passing though point A).

**Table 2:** Descriptive statistics of rigid body transformation analysis results. Measures in absolute value are indicated as ‘abs.’.

		Pitch	Pitch Abs.	Roll	Roll Abs.	Yaw	Yaw Abs.	Tot. Ang. Displ.	Lateral	Lat. Abs.	Ant.-Post.	A-P Abs.	Vertical	Vert. Abs.	Total Trans
Maxilla	Average	−0.90	1.54	−0.06	0.93	−0.02	0.91	2.33	−0.08	1.05	−1.45	1.70	−0.78	1.14	2.58
	St.Dev.	1.65	1.06	1.21	0.75	1.20	0.76	0.91	1.28	0.70	1.87	1.64	1.37	1.08	1.70
	Median	−0.92	1.52	0.00	0.78	−0.28	0.72	2.31	−0.23	0.99	−1.27	1.39	−0.78	0.93	2.03
	IQR	2.50	1.53	1.55	0.60	1.50	0.69	1.41	1.86	0.99	2.46	2.38	1.07	0.77	1.83
Mandible	Average	0.83	1.53	0.39	0.96	0.73	1.13	2.55	−0.32	1.14	−1.26	1.49	−0.61	1.05	2.51
	St.Dev.	1.88	1.34	1.24	0.85	1.33	0.99	1.20	1.50	1.00	1.78	1.59	1.51	1.23	1.81
	Median	0.36	1.20	0.46	0.75	0.41	0.75	2.34	−0.18	0.93	−0.84	0.99	−0.26	0.71	2.02
	IQR	2.59	1.52	1.04	0.72	2.00	1.38	1.30	1.54	0.91	1.41	1.18	1.05	1.20	1.35

**Table 3:** Descriptive statistics of cephalometric analysis results. Measures in absolute value are indicated as ‘abs.’.

	A/Sag	A/Sag Abs.	B/Sag	B/Sag Abs.	Pog/Sag	Pog/Sag Abs.	Δ Go/Sag	Δ Go/Sag Abs.	UIs/Sag	UIs/Sag Abs	LIs/Sag	LIs/Sag Abs	Δ U3/Sag	Δ U3/Sag Abs	Δ U6/Sag	Δ U6/Sag Abs
Average	0.52	0.96	0.86	1.31	1.00	1.67	−0.56	2.40	0.88	1.20	0.71	1.02	0.17	2.89	−0.39	2.10
St.Dev.	1.17	0.83	1.36	0.91	1.91	1.33	3.26	2.23	1.30	0.99	1.29	1.04	3.55	1.97	2.70	1.68
Median	0.30	0.83	1.10	1.19	1.04	1.50	−0.48	1.35	0.71	1.02	0.44	0.58	0.12	2.78	−0.54	1.65
IQR	1.30	1.15	1.41	1.12	2.38	2.16	2.65	2.90	1.30	1.01	0.82	1.01	5.40	3.58	3.09	1.85

**Table 4:** Clinically significant Spearman’s correlation coefficients extrapolated from the correlation matrix.

Correlated Values			Correlated Values			Correlated Values		
Mx pitch	corr. coeff.	−0.008	Md pitch	corr. coeff.	−0.037	Mx yaw	corr. coeff.	0.613
A/Sag	Sig.2-tail	0.97	B/Sag	Sig.2-tail	0.871	Md yaw	Sig.2-tail	0.002
Mx pitch	corr. coeff.	−0.221	Md pitch	corr. coeff.	−0.167	Mx lat	corr. coeff.	0.581
B/Sag	Sig.2-tail	0.323	Pog/Sag	Sig.2-tail	0.459	Md lat	Sig.2-tail	0.005
Mx pitch	corr. coeff.	−0.317	Md pitch	corr. coeff.	0.084	Mx a-p	corr. coeff.	0.819
Pog/Sag	Sig.2-tail	0.151	Δ Go/Sag	Sig.2-tail	0.71	Md a-p	Sig.2-tail	0
Mx pitch	corr. coeff.	0.018	Md pitch	corr. coeff.	0.169	Mx vert	corr. coeff.	0.648
Δ Go/Sag	Sig.2-tail	0.938	UIs/Sag	Sig.2-tail	0.453	Md vert	Sig.2-tail	0.001
Mx pitch	corr. coeff.	0.318	Md pitch	corr. coeff.	−0.109	Mx pitch	corr. coeff.	−0.461
UIs/Sag	Sig.2-tail	0.149	Lis/Sag	Sig.2-tail	0.629	Md a-p	Sig.2-tail	0.031
Mx pitch	corr. coeff.	−0.053	Md pitch	corr. coeff.	−0.012	Mx pitch	corr. coeff.	−0.522
Lis/Sag	Sig.2-tail	0.816	Δ U3/Sag	Sig.2-tail	0.958	Md vert	Sig.2-tail	0.013
Mx pitch	corr. coeff.	0.22	Md pitch	corr. coeff.	0.047	Md pitch	corr. coeff.	−0.542
Δ U3/Sag	Sig.2-tail	0.326	Δ U6/Sag	Sig.2-tail	0.836	Mx A-P	Sig.2-tail	0.009
Mx pitch	corr. coeff.	0.307	Mx pitch	corr. coeff.	0.523	Md pitch	corr. coeff.	−0.612
Δ U6/Sag	Sig.2-tail	0.165	Md pitch	Sig.2-tail	0.012	Mx vert	Sig.2-tail	0.002
Md pitch	corr. coeff.	0.02	Mx roll	corr. coeff.	0.729	A/McN	corr. coeff.	−0.519
A/Sag	Sig.2-tail	0.93	Md roll	Sig.2-tail	0	Inc. Prot.	Sig.2-tail	0.013

## Data Availability

The complete collected data is available as supplementary material.

## Supplementary Materials

The following are available online at https://www.mdpi.com/article/10.3390/jpm11111237/s1. Complete Dataset and Class II vs Class III compar.
